# The 2-aminoethoxydiphenyl borate analog, DPB161 blocks store-operated Ca^2+^ entry in acutely dissociated rat submandibular cells

**DOI:** 10.18632/oncotarget.18623

**Published:** 2017-06-27

**Authors:** Kunkun Xia, Zegang Ma, Jianxin Shen, Menghan Li, Baoke Hou, Ming Gao, Shuijun Zhang, Jie Wu

**Affiliations:** ^1^ Department of Hepatobiliary and Pancreatic Surgery, First Affiliated Hospital of Zhengzhou University, Zhengzhou, China; ^2^ Department of Neurobiology, Barrow Neurological Institute, St. Joseph’s Hospital and Medical Center, Phoenix, AZ, USA; ^3^ Department of Physiology, Shandong Provincial Key Laboratory of Pathogenesis and Prevention of Neurological Disorders, Shandong Provincial Collaborative Innovation Center for Neurodegenerative Disorders and State Key Disciplines, Physiology, Medical College of Qingdao University, Qingdao, China; ^4^ Department of Physiology, Shantou University Medical College, Shantou, China

**Keywords:** 2-aminoethoxydiphenyl borate, store-operated Ca^2+^ entry (SOCE), DPB161, Ca^2+^ oscillations, rat submandibular cells

## Abstract

Cellular Ca^2+^ signals play a critical role in cell physiology and pathology. In most non-excitable cells, store-operated Ca^2+^ entry (SOCE) is an important mechanism by which intracellular Ca^2+^ signaling is regulated. However, few drugs can selectively modulate SOCE. 2-Aminoethoxydiphenyl borate (2APB) and its analogs (DPB162 and DPB163) have been reported to inhibit SOCE. Here, we examined the effects of another 2-APB analog, DPB161 on SOCE in acutely-isolated rat submandibular cells. Both patch-clamp recordings and Ca^2+^ imaging showed that upon removal of extracellular Ca^2+^ ([Ca^2+^]_o_=0), rat submandibular cells were unable to maintain ACh-induced Ca^2+^ oscillations, but restoration of [Ca^2+^]_o_ to refill Ca^2+^ stores enable recovery of these Ca^2+^ oscillations. However, addition of 50 μM DPB161 with [Ca^2+^]_o_ to extracellular solution prevented the refilling of Ca^2+^ store. Fura-2 Ca^2+^ imaging showed that DPB161 inhibited SOCE in a concentration-dependent manner. After depleting Ca^2+^ stores by thapsigargin treatment, bath perfusion of 1 mM Ca^2+^ induced [Ca^2+^]_i_ elevation in a manner that was prevented by DPB161. Collectively, these results show that the 2-APB analog DPB161 blocks SOCE in rat submandibular cells, suggesting that this compound can be developed as a pharmacological tool for the study of SOCE function and as a new therapeutic agent for treating SOCE-associated disorders.

## INTRODUCTION

Intracellular Ca^2+^ ([Ca^2+^]_i_) signals play critical roles in the modulation of cellular physiology and pathophysiology. In many non-excitable cells, intracellular Ca^2+^ signals respond to extracellular agonist stimulation in an oscillatory, rather than sustained, manner [1–3]. It is well accepted that two distinct intracellular Ca^2+^ pools, the inositol-1, 4, 5-trisphosphate (InsP_3_)-sensitive and ryanodine-sensitive pools, participate in the genesis of oscillatory Ca^2+^ signals [4]. In 1986, Putney presented a model for capacitive calcium (Ca^2+^) entry conveying that depletion of endoplasmic reticulum-stored Ca^2+^ leads to activation of plasma membrane Ca^2+^ channels that mediate influx of Ca^2+^ from the extracellular space into cells [5], in a process called store-operated Ca^2+^ entry (SOCE). Recent molecular studies have identified that stromal interaction molecule (STIM), the Ca^2+^ sensor of the intracellular compartments, together with Orai, the subunits of Ca^2+^ permeable channels on the plasma membrane [6], cooperate in regulating multiple cellular functions as diverse as proliferation, differentiation, migration and gene expression, demonstrating that SOC channels are important targets for normal cell function and a variety of diseases [7]. Unfortunately, there are few selective blockers for SOCE. There is a need to develop new compounds for SOCE as pharmacological tools for investigation and as therapeutic agents for diseases.

**Figure 1:**
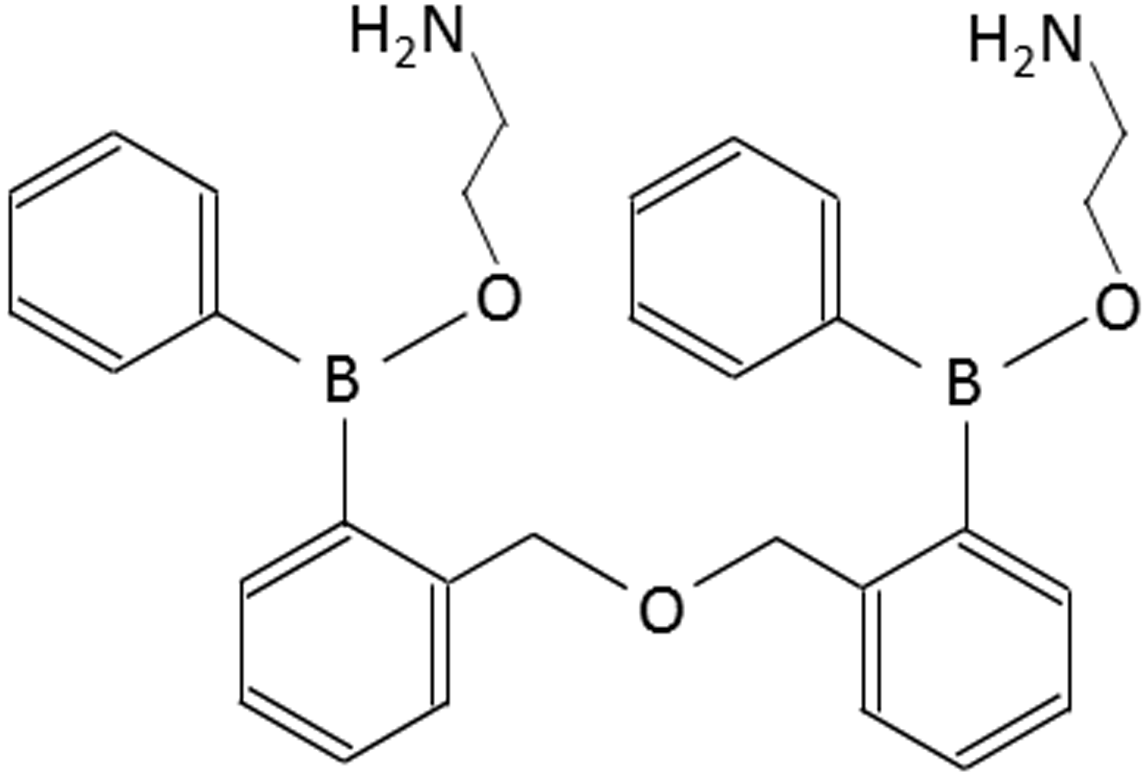
Chemical structure of DPB-161

The membrane-penetrable compound, 2-aminodiphenyl borinate (2-APB) was initially developed as membrane-permeable InsP_3_ receptor antagonist since 2-APB produced concentration-dependent inhibition of InsP_3_-induced Ca^2+^ release from mouse cerebellar membranes without affecting InsP_3_ binding [8]. After this initial report, numerous studies have reported that 2-APB antagonizes SOCE in different cell types [9, 10]. Thereafter, Mikoshiba’s group further developed 2-APB analogs and demonstrated that 2-APB analogs exhibit more selective inhibition of SOCE compared to inhibition of InsP_3_-induced Ca^2+^ release, in either transfected cell lines or DT40 cells [11, 12]. In the present study, we used patch-clamp recording and Ca^2+^ imaging to test the effects of a novel 2-APB analog, DPB161 (Figure 1) on SOCE in rat submandibular cells. We found that DPB161 inhibited SOCE in a concentration-dependent manner. Thus, this study provides evidence that a 2-APB analog, DPB161 serves as a SOCE blocker to modulate cell Ca^2+^ signals in rat submandibular cells.

## RESULTS

### Rat submandibular cells model of SOCE

**Figure 2:**
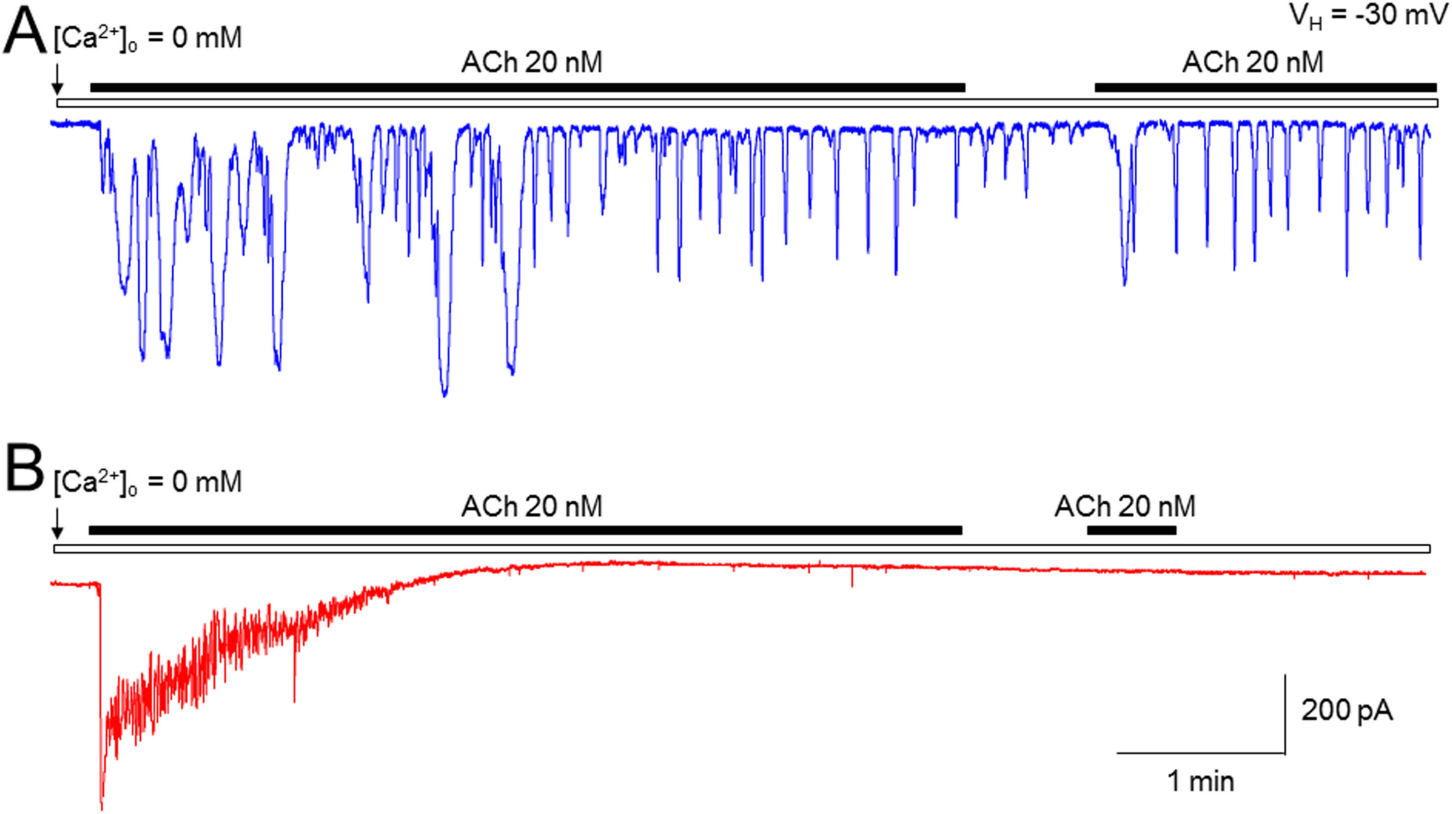
Comparison of ACh-induced Ca2+ oscillations between pancreatic acinar cells and submandibular cells using patch-clamp recordings Under [Ca^2+^]_o_ = 0 condition, ACh (20 nM) induced a long-lasting Ca^2+^ oscillatory response in mouse pancreatic acinar cells **(A)** but induced a short-lasting Ca^2+^ oscillatory response in rat submandibular cells **(B)**. Typical traces from 6 cells tested are presented in A (mouse pancreatic acinar cells) and B (rat submandibular cells), respectively.

To open SOC channels, intracellular Ca^2+^ store need to be depleted. Initial experiments were designed to compare two types of cells (mouse pancreatic acinar cells and rat submandibular cells) for agonist-induced emptying of Ca^2+^ stores. Under free extracellular Ca^2+^ ([Ca^2+^]_o_=0) conditions, continuous bath-application of ACh (20 nM) induced persistent Ca^2+^ oscillatory responses as measured by the Ca^2+^-dependent Cl^-^ current using patch-clamp whole-cell recordings in mouse pancreatic acinar cells (Figure 2A). However, in rat submandibular cells under the same experimental conditions, ACh-induced Ca^2+^ oscillatory responses stopped within minutes, and after washout of ACh with [Ca^2+^]_o_=0 solution for minutes, ACh still failed to induce any oscillatory responses (Figure 2B), suggesting an emptied intracellular Ca^2+^ store that was not refilled with extracellular Ca^2+^ in a SOCE-dependent manner. These results demonstrate that compared to pancreatic acinar cells, the response of rat submandibular cells to ACh stimulation is more dependent on Ca^2+^ entering through SOC channels to refill the Ca^2+^ store. Therefore, we used rat submandibular cells for all further experiments.

### DPB161 blocks Ca^2+^ store refilling following ACh-induced depletion of intracellular Ca^2+^ stores

**Figure 3:**
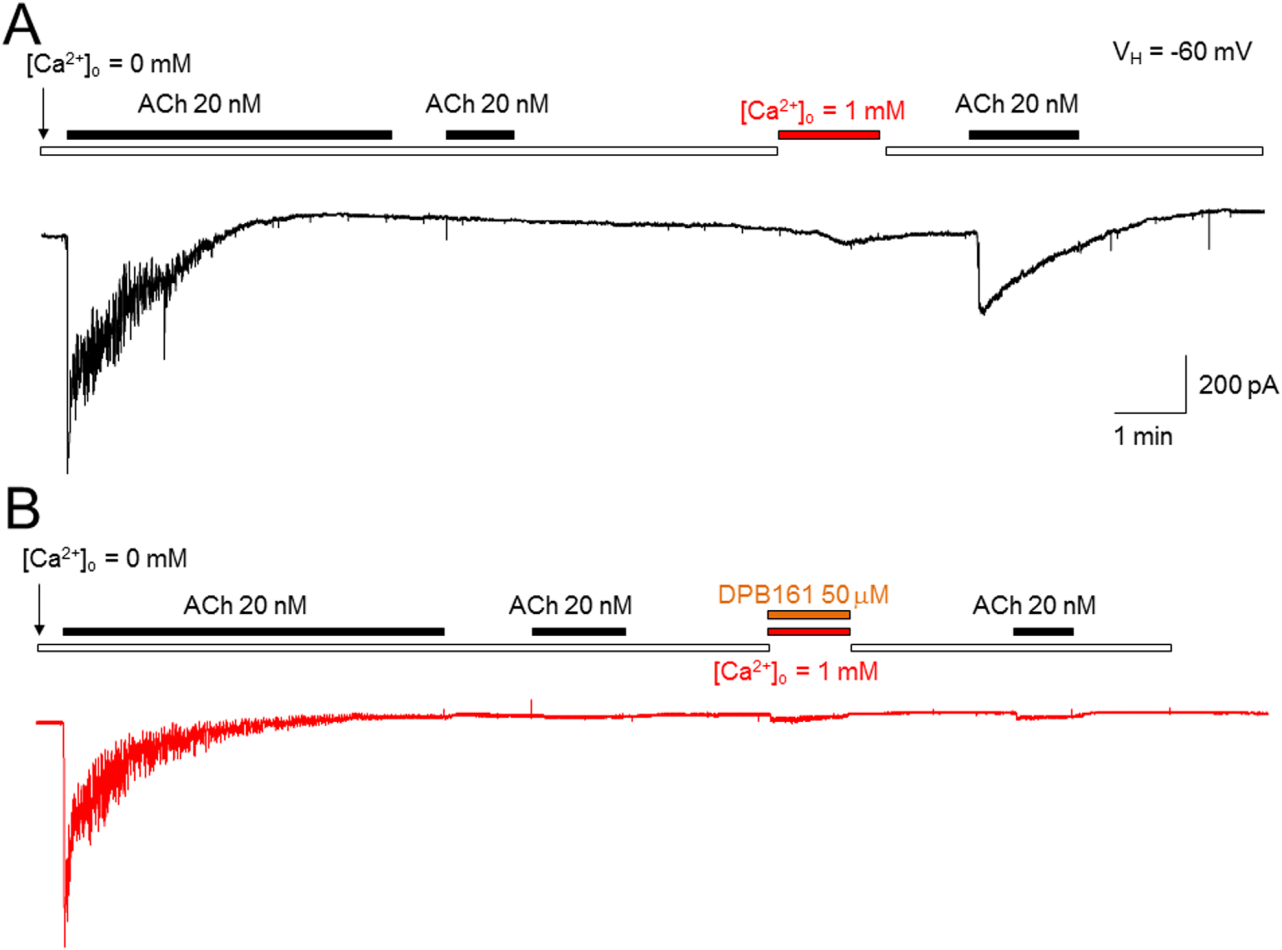
Effects of DPB-161 on [Ca^2+^]_i_ responses to ACh measured by patch-clamp recordings in rat submandibular cells **(A)** Under [Ca^2+^]_o_ = 0 conditions, bath-application of 20 nM ACh induced Ca^2+^oscillatory response. Bath-application of 1 mM Ca^2+^ refills the Ca^2+^stores through opened SOC channels. **(B)** Using the same experimental protocol but refilling Ca^2+^ stores with 1 mM Ca^2+^ and 50 μM DBP161 prevented the refilling of Ca^2+^ stores, suggesting that DPB161 blocks SOCE. Typical traces in A and B are presented from 6 submandibular cells tested, respectively.

In these experiments, we characterized the effects of DPB161 on SOCE-dependent intracellular Ca^2+^ store refilling in rat submandibular cells. Under [Ca^2+^]_o_=0 conditions, bath-application of 20 nM ACh for 3-5 min induced an oscillatory response, which likely caused complete release of Ca^2+^ from the intracellular Ca^2+^ store since after washout of ACh for one min, reapplication of ACh failed to induce any oscillatory responses. Then, 1 mM Ca^2+^ was bath-applied to refill the Ca^2+^ store. Thereafter, we applied ACh again under [Ca^2+^]_o_=0 conditions and induced a Ca^2+^ response, suggesting that under [Ca^2+^]_o_=0 condition, the initial 20 nM ACh application emptied the Ca^2+^ store, and the subsequent bath-perfusion of 1 mM [Ca^2+^]_o_ successfully refilled Ca^2+^ store (Figure 3A). When 50 μM DPB161 was included in the bath-perfusion with 1 mM [Ca^2+^], refilling of the Ca^2+^ store as prevented. Similar experimental results were obtained from 6 cells tested. These results suggest that DPB161 blocks SOCE (Figure 3B).

### DPB161 blocks Ca^2+^ store refilling measured by fura-2 Ca^2+^ imaging

**Figure 4:**
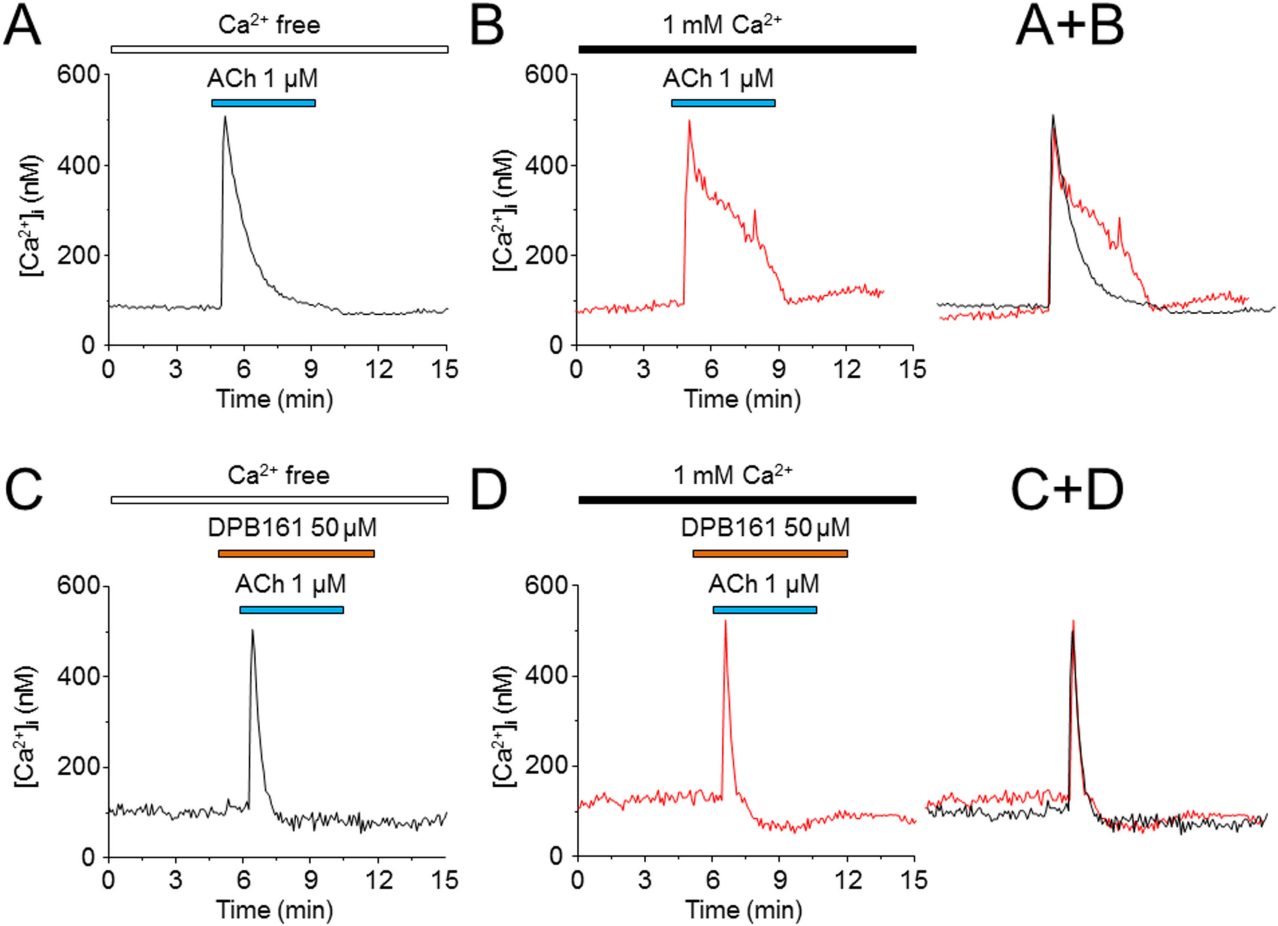
Effects of DPB-161 on [Ca^2+^]_i_ responses to ACh measured by Ca^2+^ imaging in rat submandibular cells **(A)** In the presence of extracellular Ca^2+^, the cell was stimulated with 1 μM ACh (4 min). **(B)** ACh was applied to the cell in a Ca^2+^-free solution. **(C)** Before (1 min) and during stimulation with ACh in the Ca^2+^-free solution, 50 μM DPB-161 was applied. **(D)** Before (1 min) and during stimulation with ACh in the Ca^2+^-containing solution, 50 μM DPB-161 was applied. The representative trace of 11-16 experiments is shown in each panel.

To further evaluate the role of DPB161 in inhibiting SOCE, we measured intracellular Ca^2+^ concentrations ([Ca^2+^]_i_) using Fura-2 imaging in isolated rat submandibular cells. Application of high concentration (1 μM) of ACh resulted in a spike in [Ca^2+^]_i_ that declined much faster under [Ca^2+^]_o_=0 conditions (Figure 4A) than under 1 mM [Ca^2+^]_o_ conditions (Figure 4B), suggesting that ACh empties Ca^2+^ stores and opens SOCE to refill the Ca^2+^ stores. However, when the bath solution contained DPB161 (50 μM), 1 μM ACh induced [Ca^2+^]i spike, with the same rise and decline, regardless of whether the bath solutions contained Ca^2+^ or not (Figure 4C, 4D). Statistical analysis indicated that the ACh-induced current areas (between 4 – 12 min) were 4504.8 ± 409.4 under 1 mM [Ca^2+^]_o_ condition (n=12, A) and 3542.4 ± 231.4 for the [Ca^2+^]_o_=0 conditions (n=13, B). The difference was significant (*p*<0.05, unpaired t-test). In the presence of 50 μM DPB161, the ACh-induced current areas under 1 mM [Ca^2+^]_o_ (n=11, C) and [Ca^2+^]_o_=0 conditions (n=16, D) were 1575.4 ± 80.2 and 1377.8 ± 190.8 (*p*>0.05, unpaired t-test). These results support the patch-clamp finding that DPB161 blocks the refilling of the Ca^2+^ store through SOCE channels.

### DPB161 blocks Ca^2+^ store refilling in a concentration-dependent manner

**Figure 5:**
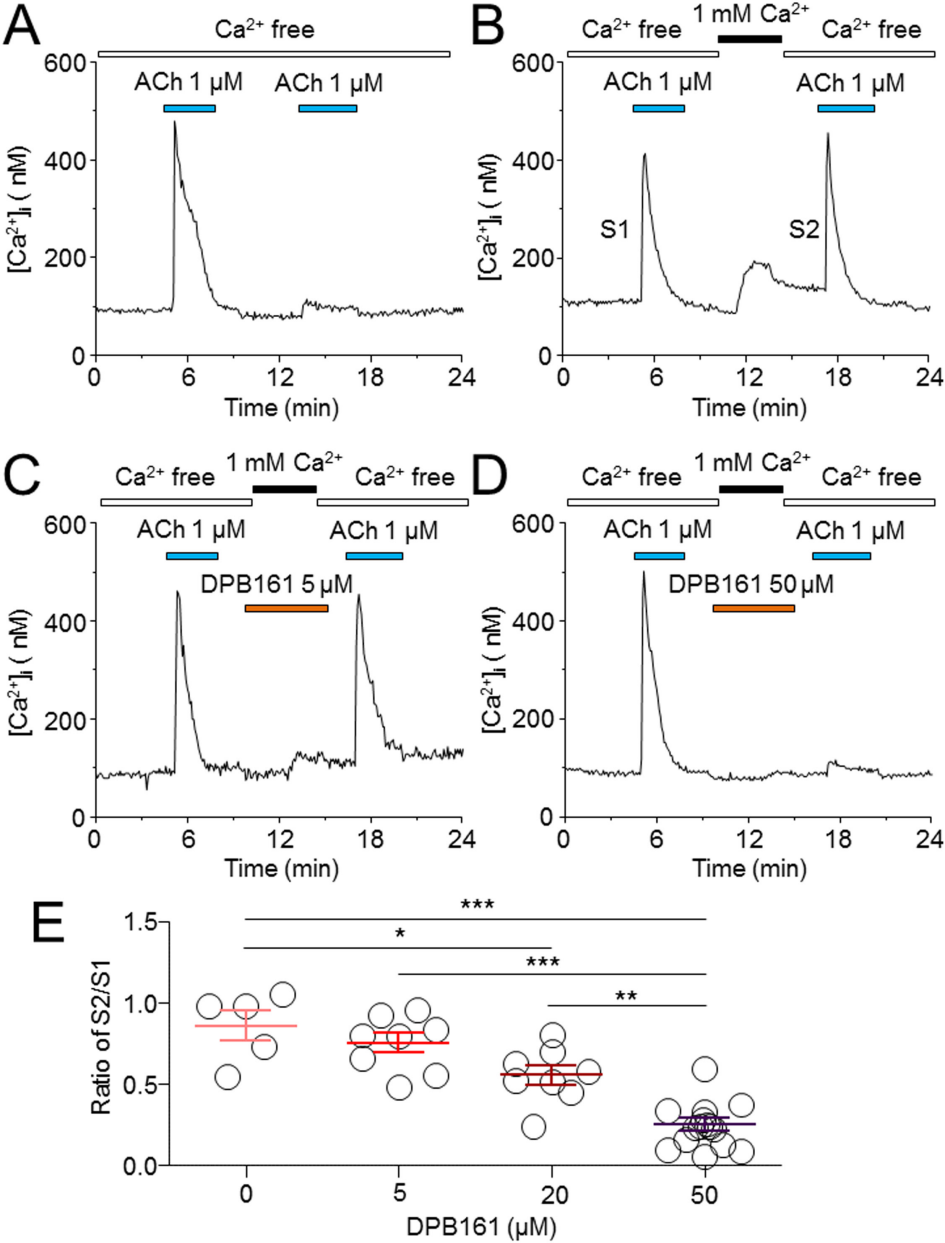
Effects of DPB-161 on Ca^2+^ store refilling following ACh-induced Ca^2+^ store depletion in rat submandibular cells **(A)** In Ca^2+^-free solution, the cell was stimulated by 1 μM ACh (4 min). After the first challenge of ACh, a second ACh challenge (4 min) was performed. **(B)** Between the two ACh challenges, Ca^2+^-containing solution was applied for 2 min. **(C)** Before (1 min) and during addition of extracellular Ca^2+^, 5 μM DPB-161 was applied. **(D)** Before (1 min) and during addition of extracellular Ca^2+^, 50 μM DPB-161 was applied. The representative trace of 5-19 experiments is shown in each panel. **(E)** Concentration-response relationships for DPB-161 inhibition of store-operated Ca^2+^ entry. Relative sizes of the second ACh responses to the first ones are shown, measured by the area under the curve. Values are means of 5, 8, 8, and 15 cells tested for 0, 5, 20 and 50 μM DPB-161, respectively. Vertical bars indicate SE. Statistical significance was obtained by one-way ANOVA. *: *p* < 0.05, **: *p* < 0.01, ***: *p* < 0.001.

To compare the effects of different concentrations of DPB161 on SOCE, we quantitatively measured ACh (1 μM)-induced elevation of [Ca^2+^]_i_ twice with 5-10 min intervals with or without external Ca^2+^. As shown in Figure 5A, under [Ca^2+^]_o_=0 without external Ca^2+^ refilling, the second ACh-induced elevation of [Ca^2+^]_i_ was eliminated, suggesting that the first ACh exposure (1 μM for ∼4 min) has already depleted the Ca^2+^ store. However, with [Ca^2+^]_o_ perfusion (1 mM for ∼ 4 min), the Ca^2+^ store was able to refill as the second ACh exposure induced [Ca^2+^]_i_ elevation to a similar extent (Figure 5B). Thereafter, we used this protocol to compare the ratio of the second [Ca^2+^]_i_ response (S2) and first [Ca^2+^]_i_ response with different concentrations of DPB161 under conditions of 1 mM [Ca^2+^]_o_ perfusion. One-way ANOVA showed a statistical difference in S2/S1 ratio after perfusion of DPB161 (F_(4)_=56.1, *p*<0.001). Tukey analysis showed little effect of 5 μM DPB161 in prevention of [Ca^2+^]_o_ entry compared to 0 μM DPB161 (without DPB161, the S2/S1 ratio was normalized as 100%, the 5 μM DPB161 was 87.6±7.1%, *p*>0.05, n=8, Figure 5C), but 20 μM DPB161 reduced S2/S1 ratio to 64.6±7.0% (*p*<0.001, n=8), and 50 μM DPB161 reduced S2/S1 ratio to 23.0±4.8% (*p*<0.001, n=15, Figure 4D). These data demonstrate that DPB161 prevents SOCE in a concentration-dependent manner in rat submandibular cells (Figure 5E).

### DPB161 blocks SOC channel-dependent [Ca^2+^]_i_ elevation

**Figure 6:**
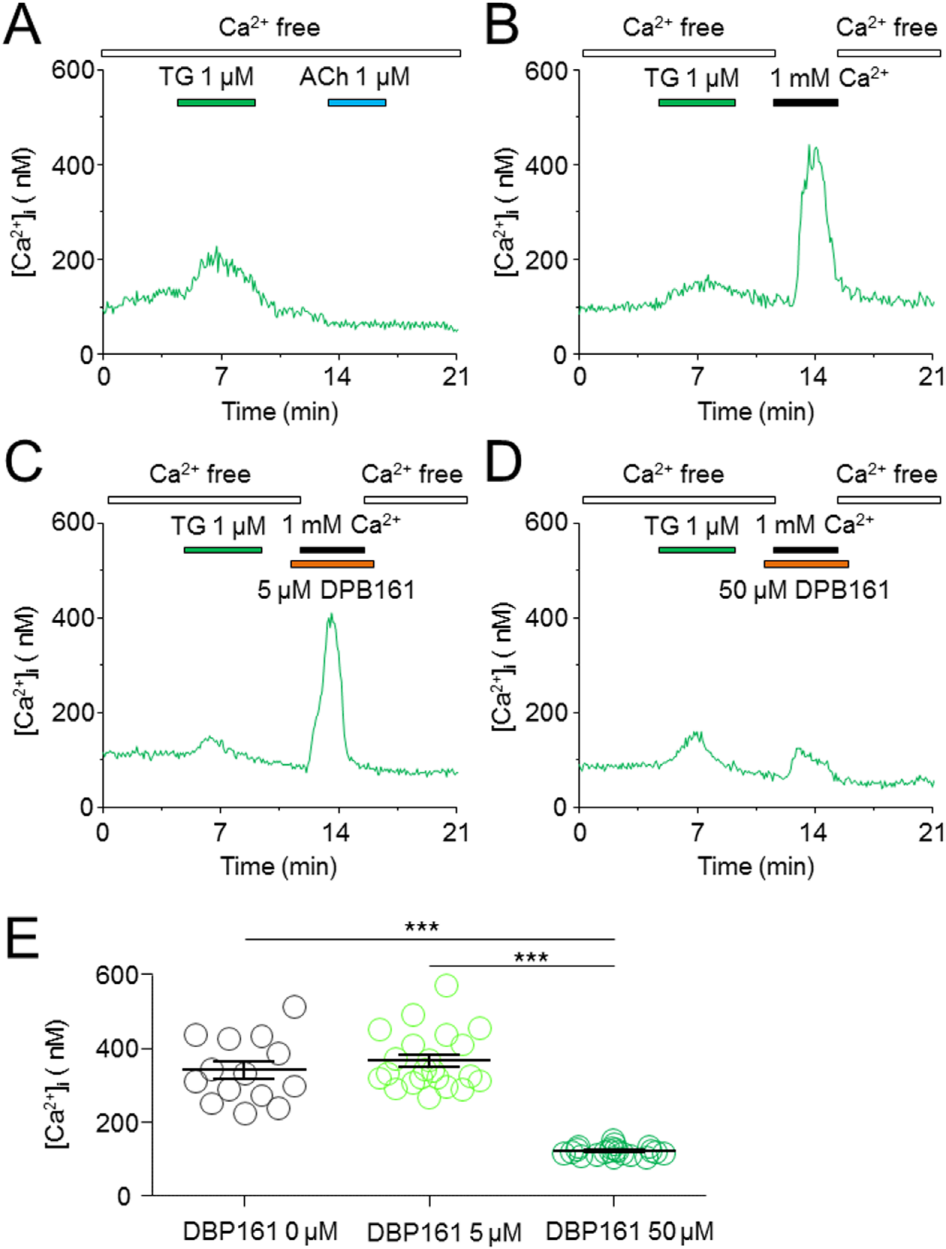
Effects of DPB-161 on the Ca^2+^ entry following thapsigargin-induced Ca^2+^ store depletion in rat submandibular gland acinar cells **(A)** In Ca^2+^-free solution, 1 μM thapsigargin (TG) was first applied (4 min), followed by 1 μM ACh. **(B)** After TG treatment (4 min), the Ca^2+^-containing solution was applied (2 min). **(C)** Before (1 min) and during addition of extracellular Ca^2+^, 5 μM DPB-161 was applied. **(D)** Before (1 min) and during addition of extracellular Ca^2+^, 50 μM DPB-161 was applied. **(E)** Statistical analysis of 14-21 experiments are shown in panels B, C, D.

Data presented thus far demonstrates that DPB161 blocks ACh-triggered SOCE in rat submandibular cells. Finally, we directly measured SOC channel-mediated [Ca^2+^]_i_ elevation under conditions of DPB161 treatment. To measure SOC channel-mediated [Ca^2+^]_i_ elevation, we depleted intracellular Ca^2+^ store by pre-treating cells with thapsigargin (TG) under [Ca^2+^]_o_=0 conditions. Depletion of Ca^2+^ stores was demonstrated by the inability of ACh application to induce [Ca^2+^]_i_ elevation (Figure 6A). After depletion of the Ca^2+^ store, SOC channels were opened, and we were able to record 1 mM external Ca^2+^-induced [Ca^2+^]_i_ elevation through SOC channels (Figure 6B). Then, we tried to refill the Ca^2+^ store with 1 mM [Ca^2+^]_o_ in the presence of 5 μM (Figure 6C) or 50 μM DPB161 (Figure 6D). At 50 μM, but not 5 μM, DPB161 prevented the SOC channel-mediated [Ca^2+^]_i_ elevation.

## DISCUSSION

In the present study, we employed both patch-clamp recordings and Fura-2 Ca^2+^ imaging to examine the effects of a 2-APB analog, DPB161 on SOCE in freshly isolated rat submandibular cells. We demonstrate that DPB161 blocks rat submandibular cell SOCE in a concentration-dependent manner, suggesting that this 2-APB analog can serve as a pharmacological tool to investigate intracellular Ca^2+^ signaling mechanisms and provide a new therapeutic strategy for treatment of SOCE-associated diseases.

Since previous experiments showed that pharmacological manipulation of SOCE using 2APB analogs in different cell lines [11, 12], in our initial experiments, we sought an appropriate natural cells for studying the effects of DPB161 on SOCE. In accord with our experience of using pancreatic acinar cells [13–17], we first tested the effects of ACh-induced Ca^2+^ oscillations in mouse pancreatic acinar cells under [Ca^2+^]_o_=0 conditions, anticipating that ACh (10-20 nM)-induced Ca^2+^ oscillations world terminate in the absence of external Ca^2+^ due to an inability to refill the emptied Ca^2+^ stores resulting from persistent ACh exposure. However, our data shows that even under [Ca^2+^]_o_=0 conditions, ACh-induced Ca^2+^ oscillations continued for more than 30 min, suggesting that ACh-induced Ca^2+^ oscillations are not strongly dependent on SOCE to refill Ca^2+^ stores in pancreatic acinar cells. The precise reason is still unclear, but could be due to the highly expressed Ca^2+^ pump on the membrane of Ca^2+^ stores, which may be strong enough to reuptake released Ca^2+^ back to Ca^2+^ stores to maintain the persistent Ca^2+^ oscillations during exposure of ACh. Then, we selected rat submandibular cells based on previous work showing that ACh-induced Ca^2+^ oscillations in rat submandibular cells are dependent on external Ca^2+^ entry since ACh only induces a short-lasting Ca^2+^ oscillations if the external Ca^2+^ is removed [18, 19]. In the present study, we confirmed this phenomenon and further demonstrate the SOCE dependence of the persistent Ca^2+^ oscillations in response to ACh stimulation in rat submandibular cells. Compared to mouse pancreatic acinar cells, rat submandibular cells exhibit a much shorter duration of Ca^2+^ oscillation in response to persistent ACh exposure under [Ca^2+^]_o_=0 conditions, suggesting that rat submandibular cells are an excellent cell model to evaluate the pharmacological effects of agents that modulate SOCE.

Under [Ca^2+^]_o_=0 conditions, we exposed cells to either ACh or thapsigargin to deplete Ca^2+^ stores and open SOC channels, and tested the effects of DPB161 on SOCE. Patch-clamp experiments showed that persistent exposure of ACh (20 nM) induces a short-lasting oscillatory response (measured Ca^2+^-dependent Cl^-^ currents) under [Ca^2+^]_o_=0 conditions. This is due to the ACh has released all releasable Ca^2+^ from Ca^2+^ stores without extracellular Ca^2+^ refilling. After emptying Ca^2+^ stores, exposure to 1 mM external Ca^2+^ enables the refilling of Ca^2+^ stores and recovery of the 20 nM ACh-induced oscillatory responses (Figure 3A). We used this experimental protocol to examine the effect of DPB161 on Ca^2+^ refilling, and found that co-application of 50 μM DPB161 in the presence of 1 mM external Ca^2+^ prevents the refilling of Ca^2+^ stores as judged by the lack of a detectable ACh-induced response after attempting to refill Ca^2+^ stores in the presence of 50 μM DPB161 (Figure 3B). These data suggest that 50 μM DPB161 completely blocks SOCE in rat submandibular cells. Ca^2+^ imaging experiments comparing [Ca^2+^]_i_ elevation by ACh in the presence and absence of external Ca^2+^ (1 mM) indicate that the duration of the [Ca^2+^]_i_ response is remarkably short in the absence of extracellular Ca^2+^, suggesting that during ACh depletion of Ca^2+^ stores, the refilling of Ca^2+^ stores requires the presence of external Ca^2+^ (Figure 4A, 4B). Using the same protocol, but comparing 1 mM external Ca^2+^ plus 50 μM DPB161 perfusion vs. perfusion in the absence of external Ca^2+^, we find ACh induces similar waveforms of [Ca^2+^]_i_ elevation when perfused with 50 μM DPB161 plus 1 mM external Ca^2+^, suggesting that DPB161 blocks SOCE. To qualitatively compare the concentration dependence of DPB161 on SOCE, we applied ACh twice under conditions with and without external Ca^2+^. Without external Ca^2+^, the second Ca^2+^ response is dramatically reduced (Figure 5A), whereas in the presence of external Ca^2+^, sequential applications of ACh (interval > 10 min) induces [Ca^2+^]_i_ elevations of similar size (Figure 5B). In the presence of different concentrations of DPB161 during the second ACh application, the concentration-dependent reduction in the ratio of the second to the first peak amplitude of ACh-induced [Ca^2+^]_i_ elevations under free extracellular Ca^2+^ conditions demonstrates that DPB161 blocks SOCE in a concentration-dependent manner (Figure 5E). Under [Ca^2+^]_o_ =0 conditions, thapsigargin depletes Ca^2+^ stores and opens SOC channels enabling bath perfusion with 1 mM Ca^2+^ to increase [Ca^2+^]_i_ through SOC channels. Co-application of DPB161 with 1 mM Ca^2+^ prevents SOCE-mediated [Ca^2+^]_i_ elevation. Taken together, these results suggest that the 2APB analog, DPB161 inhibits SOCE in rat submandibular cells.

Previous work demonstrates that the 2APB analogs DPB162 and DPB163 potently block SOCE in cell lines, in which these 2-APB analogs show high affinity for blocking the SOCE [12, 20, 21]. We further demonstrate that another 2APB analog, DPB161 also inhibits SOCE in rat submandibular cells. However, the potency of SOCE inhibition by DPB161 in rat submandibular cells is lower. For example, 5 μM DPB161 fails to block SOCE elicited by ACh- or thapsigargin-induced depletion of Ca^2+^ stores. This suggests that the structure difference between DPB161 and the other 2APB analogs may partially explain the difference in its potency to block the SOCE. The IC_50_ values for SOCE inhibition by DPB161 are about 50-fold higher than DPB163 (1.7x10^-1^ vs. 5.9x10^-2^ μM) in InsP_3_ receptor-deficient DT40 cells [11], suggesting a lower affinity of DPB161 compared to DPB163 for inhibition of SOCE, which is consistent with the findings in the present study. However, the affinity of DPB161 for inhibition of the SOCE in native rat submandibular cells is much lower than that in InsP_3_ receptor-deficient DT40 cells. These lines of evidence suggest a diversity of SOCE in different cell types.

## MATERIALS AND METHODS

All experiments were performed in accordance with approved guidelines set and all experimental protocols were approved by the First Affiliated Hospital of Zhengzhou University, the Barrow Neurological Institute, and the Shantou University Medical College Ethical Committees.

### Preparation of single rat submandibular cells

Acute dissociation of rat submandibular cells was performed as described in our previous publication [19]. In brief, after isoflurane anesthesia, the fragments from adult Wistar rats (both genders) submandibular glands were minced and digested with collagenase (100 units/ml) for 15 min at 37°C. At the end of the collagenase digestion, the cell suspension was gently pipetted to obtain further separation of the cells. The cell suspension was then filtered through a 100 pm platinum mesh, washed twice with the extracellular solution containing 0.2% bovine serum albumin (BSA), and kept in the solution until use. A small amount of the cell suspension was added to a 2 ml-capacity experimental bath on a stage of an inverted microscope (Olympus IX7, Japan). The isolated cells usually adhered to the bottom within 15–20 min and were used for recording within 3 h after preparation. All experiments were performed at room temperature (22 ± 1°C). For the experiments, single cells were usually chosen, but in some cases, small clusters of three to five cells were also used.

### Patch-clamp whole-cell recordings

Conventional whole-cell, patch-clamp recording was used to record the Ca^2+^-activated Cl^−^ currents for monitoring intracellular Ca^2+^ signal oscillations, as reported previously [13, 17, 19]. The recording pipettes, made from borosilicate glass capillaries (Narishige, Japan), were pulled in two stages by a vertical microelectrode puller (P10, Narishige, Japan) to make patch-clamp electrodes. The tip resistance of the electrodes ranged from 4-6 MΩ when filled with 140 mM KC1-containing internal solution. After reaching a high seal resistance (> 2 GΩ), the whole-cell configuration was established by applying a brief negative suction. Transmembrane currents were recorded with a patch-clamp amplifier (Axopatch 200B; Molecular Devices; Sunnyvale, CA USA) at a holding potential (V_H_) of −60 mV. No series resistance was compensated in these experiments.

### Measurement of intracellular Ca^2+^ concentration ([Ca^2+^]_i_)

Isolated cells were preloaded with a standard external solution containing 2 μM Fura-2/AM (Invitrogen, Oregon, USA) for 40 min at 37°C. Ca^2+^ images were captured using an inverted fluorescence microscope (Olympus IX70, Japan) and a silicon intensifier target camera (Cool SNAPfx, Roper Scientific), and recorded on a fluorescence-imaging system (LAMBAD 10-2, Sutter Instrument Co, USA). For measurement of [Ca^2+^]_i_ with Fura-2, the excitation wavelengths were 340 nm and 380 nm, a dichroic mirror of 440 nm and an emission filter of 510 nm were used. [Ca^2+^]_i_ was calculated from the ratio (R) of the two fluorescence intensities, F340/F380 [22]. The change in [Ca^2+^]_i_ was calculated by subtracting [Ca^2+^]_i_ at the peak response level from that at the pre-stimulated level.

### Experimental solutions

The standard extracellular (bath) solution contained (in mM) NaCl 130, KCl 14.7, CaC12 1.0, MgC12 1.13, HEPES 10, and glucose 10, titrated with NaOH to pH 7.2. To make a Ca^2+^-free external solution ([Ca^2+^]_o_ = 0 mM), CaCl_2_ was removed from and 1 mM EGTA was routinely added to the standard extracellular solution. The standard intracellular (pipette) solution contained (in mM) KC1 140, MgCl_2_ 1.13, HEPES 10, glucose 10, ATP 1.0, and EGTA 0.25, titrated with KOH to pH 7.2.

### Application of drugs

Cells were perfused continuously with a stream of the extracellular solution (∼0.5 ml/min). A computer-controlled U-tube system was used for drug application (Huang et al., 2016). The reagents used for the present study were acetylcholine and thapsigargin (Sigma-Aldrich, St. Louis, MO), collagenase (Wako Chemicals, Japan), and Fura-2/AM (Invitrogen, Oregon, USA). The 2-APB analog DPB161 (Figure 1) was a gift from Professor Katsuhiko Mikoshiba.

### Data analysis and statistics

For patch-clamp experiments, the Ca^2+^-activated Cl^-^ current responses were presented as net charge (current area/Cm/min), then the drug-induced changes were normalized to the current response (induced by ACh) before testing drug (e.g., DPB161) exposure (Baseline). If data were obtained from the same recorded cell and the changes of ACh response were compared prior to, during and after testing drug exposure, a paired t-test was used. To analyze multiple effects (e.g., different concentrations), one-way ANOVA with Tukey’s post hoc tests were used.
